# Inclusion of Control Data in Fits to Concentration–Response Curves Improves Estimates of Half-Maximal Concentrations

**DOI:** 10.1021/acs.jmedchem.3c00107

**Published:** 2023-09-12

**Authors:** Van Ngoc Thuy La, Stanley Nicholson, Amna Haneef, Lulu Kang, David D. L. Minh

**Affiliations:** † Department of Biology, Illinois Institute of Technology, Chicago, Illinois 60616, United States; ‡ Department of Applied Mathematics, Illinois Institute of Technology, Chicago, Illinois 60616, United States; § Department of Chemistry, Illinois Institute of Technology, Chicago, Illinois 60616, United States

## Abstract

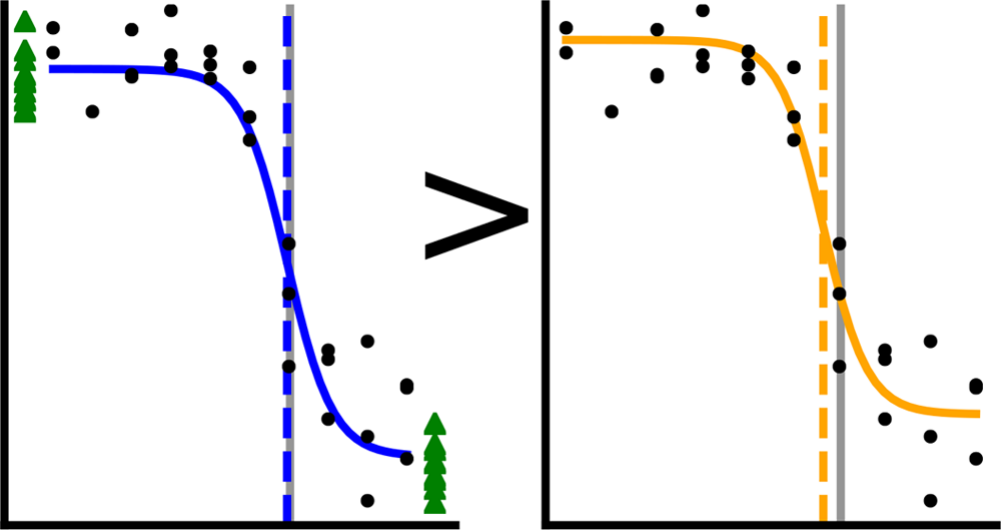

Concentration–response curves, in which the effect of varying the concentration on the response of an assay is measured, are widely used to evaluate biological effects of chemical compounds. While National Center for Advancing Translational Sciences guidelines specify that readouts should be normalized by the controls, recommended statistical analyses do not explicitly fit to the control data. Here, we introduce a nonlinear regression procedure based on maximum likelihood estimation that determines parameters for the classical Hill equation by fitting the model to both the curve and the control data. Simulations show that the proposed procedure provides more precise parameters compared with previously prescribed practices. Analysis of enzymatic inhibition data from the COVID Moonshot demonstrates that the proposed procedure yields a lower asymptotic standard error for estimated parameters. Benefits are most evident in the analysis of the incomplete curves. We also find that Lenth’s outlier detection method appears to determine parameters more precisely.

## Introduction

Some data are just underappreciated. Maybe they look different or come from a different background from most other data. Maybe they do not fit neatly into common notions of what data on a “curve” should look like. Whatever the case, they are pigeonholed into a restricted role that limits their contributions. But if people would only give them the opportunity, they could improve the entire fit.

This is the case for control data in concentration–response curves (CRCs). CRCs describe the response of a biological assay to different concentrations of a chemical compound. When the assay measures the response of an organism, the data are termed a dose–response curve. CRCs are an important step in evaluating the biological effects of a chemical compound. Widely used in pharmacology and toxicology, CRCs have become even more ubiquitous with the emergence of high-throughput screening.

CRC data are often summarized by the concentration required to induce a response halfway between the minimum and maximum. For inhibition assays, the IC_50_ specifies the half-maximal inhibitory concentration. For an agonist/stimulator assay, the EC_50_ is the concentration of substance that elicits half of the maximal response. IC_50_/EC_50_ values are often used to classify the potency of a compound against a target. For example, drugs may be categorized as high potency (below 1 μ M), medium potency (between 1 and 10 μ M), or low potency (above 10 μ M).^1^

While it is possible to analyze CRC data and determine IC_50_/EC_50_ values in many ways, standard approaches that are considered best practices in the scientific community are codified in the Assay Guidance Manual (AGM).^2^ The AGM is a free online resource written by over 100 authors and managed by the National Center for Advancing Translational Sciences with input from experts working in industry, academia, and government. Its scope includes “appropriate statistical ways to analyze assay results and accommodate minor changes to assay protocols to ensure robustness.”^3^

According to the AGM,^2^ CRC data should first be normalized by control data. As described in the section “Data Standardization for Results Management”, the specific type of control data depends on the type of assay. In enzymatic inhibition assays, negative controls contain the enzyme including cofactors and substrate(s) but no putative inhibitor. Positive controls also include substrate(s) and cofactors, but the enzyme is either absent or fully inhibited. Regardless of the assay, normalization is performed by subtracting the response by the minimum, dividing by the range (difference between the minimum and maximum), and multiplying by 100 to obtain a percentage. For most assays, the minimum is the negative control and maximum the positive control. (The exceptions are for *in vitro* functional assays of agonist and inverse agonists, in which the AGM recommends fitting to the CRC of a reference agonist to obtain the minimum and maximum.)

After normalization, the model most commonly fit to the CRC data is the classical Hill equation. As CRC data are usually monotonic with a sigmoid shape on a semilog plot, most curves are reasonably modeled by a logistic function in which the dependent variable *x* is the logarithm of the concentration *c*, *x* = log_10_(*c*/*c*^θ^), where *c*^θ^ = 1 M is the standard concentration. The classical Hill equation scales the logistic function to interpolate between the minimum response at the bottom of the curve *R*_*b*_ and maximum response at the top of the curve *R*_*t*_ as
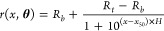
1where *x*_50_ is the base 10 logarithm of the concentration that induces the half-maximal response, *H* is the Hill slope, and **θ** = {*R*_*t*_,*R*_*b*_,*x*_50_,*H*} denotes the full set of parameters for the equation. The *x*_50_ corresponds to the base 10 logarithm of IC_50_ or EC_50_, depending on the type of assay.

While the control data are used for normalization, statistical analyses recommended by the AGM do not explicitly include control data in curve fitting. The AGM goes into considerable detail about fitting the classical Hill equation to the CRC data. Fitting to competitive binding and functional assay data is discussed in the section “Assay Operations for SAR Support”. Fitting to *in vivo* assay data is described in section 3.5 of the chapter on “*In Vivo* Assay Guidelines”. In both chapters, the AGM suggests fitting the four-parameter logistic (4PL) model, in which all of **θ** is allowed to vary, to most CRC data. The control data are not included in the fit and therefore have no effect on the estimated *x*_50_ or *H*. For some data, the AGM recommends the three-parameter logistic fixed bottom (3PLFB) model, where *R*_*b*_ is fixed at zero, or the three-parameter logistic fixed top (3PLFT) model, where *R*_*t*_ is fixed at 100. Conceptually, three-parameter models make the most sense when control data can be reasonably construed as measurements of the minimal response, *R*_*b*_ or maximal response *R*_*t*_. For example, in an enzymatic inhibition assay, if the response is proportional to the concentration of a product, then the negative control without an inhibitor is a measurement of *R*_*t*_. Fixing *R*_*b*_ or *R*_*t*_ can strongly influence the estimated values of both *x*_50_ and *H*, especially for incomplete curves.^4^ While three-parameter fits do not explicitly incorporate control data, these procedures actually give the control data a privileged position; these fits essentially set the value of a parameter based on the control as opposed to multiple concentrations along the curve.

Explicitly including control data in curve fitting, neither unduly ignoring nor privileging them, is a simple and intuitive idea. However, we found only two papers that evaluated whether it is a good idea. Weimer et al.^5^ compared various fitting procedures to androgen receptor binding assay data and to simulated data with similar parameters. While negative controls were included in all fits, positive controls were either included or excluded. They found that including positive controls in fits to experimental data most significantly changes the estimate of *R*_*b*_. In fits to simulated curves, including positive control data improved the precision of the *x*_50_ estimates (Table 2 of their paper). Thus, they recommended fits including both positive and negative controls. Kappenberg et al.^6^ considered the problem of deviating controls - when the response of the negative control deviates from the response at low concentrations. In a literature review, they found deviating controls to be a common occurrence in the toxicological literature. They found that if deviations are small, the influence of fitting with or without controls depends on whether the curves are complete, with response values near both *R*_*t*_ and *R*_*b*_. For complete curves, inclusion of controls had little influence on the fraction of estimates deemed acceptable. On the other hand, including control data was clearly beneficial for the analysis of incomplete curves.

Curve fitting including control data is only rarely mentioned in software documentation and the affiliated literature. Among commercial packages, the documentation of Origin^7^ does not mention control data whereas GraphPad documentation^8^ mentions a suggestion from Weimer et al.:^5^ Even if software is unable to accept concentrations of zero and infinity, control data may be incorporated by assigning them extremely high (for positive control data) or low (for negative control data) concentrations. The oft-cited book “Fitting models to biological data using linear and nonlinear regression: A practical guide to curve fitting”,^9^ coauthored by the founder and chief executive officer of GraphPad, spans 351 pages. As with the AGM, the use of control data is to fix curve parameters. It also describes how CRCs from control compounds may be used for global fitting and to evaluate whether parameters for a compound are different from those of the control compound. However, it does not discuss the inclusion of control conditions in curve fits. Beyond commercial software, the past decade has seen the publication of a number of papers describing software tools for fitting the Hill equation and other models to CRC data.^10−15^ These papers have focused on how the tools make curve fitting freely available and easier to use,^11,12,15^ fit data with more complex multiphasic models,^10,14^ and perform Bayesian uncertainty quantification.^13^ Other papers since 2010 have described how to make curve fitting more robust via an evolutionary algorithm,^16^ a systematic search of parameter space,^17^ or outlier detection.^18^ In only a few of these papers is fitting to control data mentioned at all. One of these mentions is in the paper about the popular *drc* package^12^ within the statistical computing environment *R*,^19^ which as of April 17, 2023 has received 1555 citations according to the Web of Science. It explains that model functions are implemented in a way such that they are defined at a concentration of zero. *eeFit* also formulates the Hill equation to avoid a singularity at zero concentration.^15^ However, these papers do not explain the benefits of including control data in curve fits.

**Figure 1:**
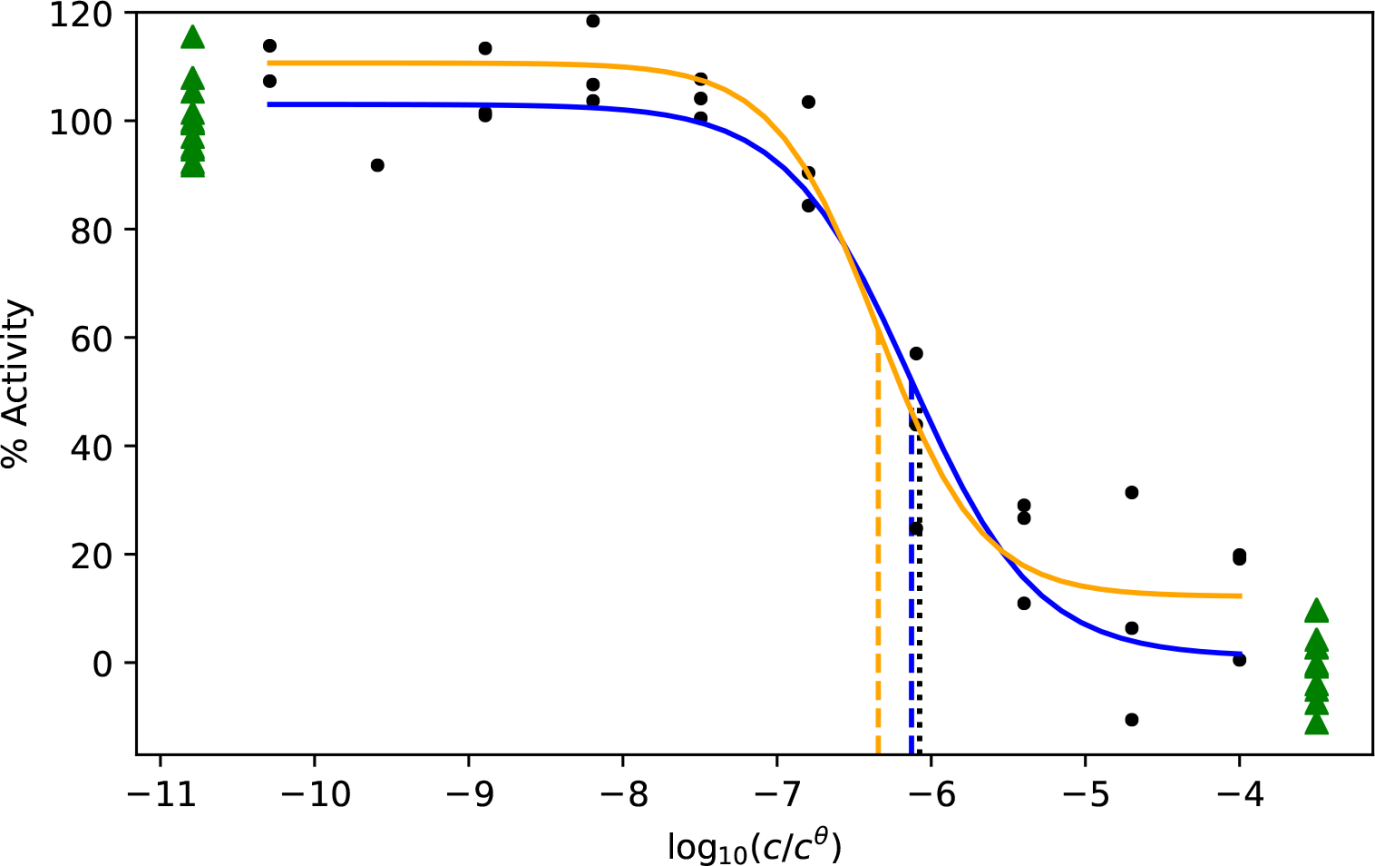
Example fits to CRC data with (blue line) or without (orange line) controls. Curve data are shown with black circles and control data as green triangles. The *x* position of the control data is not meaningful; data on the left correspond to *x* → –*∞* and data on the right correspond to *x* → *∞*. The vertical lines indicate the true *x*_50_ (black dotted) or the estimated *x*_50_ from CRC fitting with (blue dashed) or without (orange dashed) controls.

Here, we describe and evaluate a method for incorporating control data into CRC fits. Based on the assumption that measurement error is Gaussian distributed, our method is based on maximizing the likelihood of observing all of the data given the full set of parameters **θ**. We also assume homoscedasticity, that is, that all the data on the curve have the same variance, but the approach may be readily generalized to heteroscedastic data. Given that they may contain a different number of species and require a different number of dilution operations, controls are assumed to have a variance different from the curve. Without control data, the maximum likelihood estimate of model parameters is the standard procedure of nonlinear least-squares regression; it minimizes the sum of square deviation between the model and the observed data. On the other hand, when control data are included, the estimated model parameters are distinct (Figure 1) and, as we show, usually more accurate. We demonstrate the method on simulations of complete and incomplete CRCs. We also apply the proposed procedure to 1830 CRCs for two types of SARS-CoV-2 main protease enzymatic inhibition assays that were collected for the COVID Moonshot, an open-source effort to develop a COVID-19 antiviral.^20^ Lastly, we describe the results of an outlier detection method.

## Results and Discussion

As detailed in the Experimental Section, curve fitting was performed with five methods: our proposed statistical approach and up to four established procedures. Our proposed approach may be summarized as a four-parameter fitting to a logistic function with controls (4PL+C). The established procedures are a four-parameter fit without controls (4PL); a three-parameter fit with fixed bottom such that *R*_*b*_ = 0 (3PLFB); a three-parameter fit with a fixed top such that *R*_*t*_ = 100 (3PLFT); and two parameter fit with a fixed bottom and fixed top such that *R*_*b*_ = 0 and *R*_*t*_ = 100 (2PL). Simulations were analyzed with all five procedures, while biochemical assay data were analyzed with the 4PL+C, 4PL, and 3PLFB procedures. In analyses with the four established procedures, data were normalized based on the sample mean of the controls. With the proposed approach, data were normalized based on  and  obtained from the fit.

### The 4PL+C Procedure Improves the Accuracy of All Parameter Estimates Based on Simulated Data

The proposed 4PL+C procedure results in parameter estimates that have a lower error than all other tested statistical analysis procedures. For all tested procedures, the distribution of parameter estimates appears to be Gaussian, symmetric about the true value (Figures S1 and S2 of Supporting Information 1). For a set of *N* estimates of a parameter θ,  with *n* ∈ {1,2,3, ...,*N*}, the mean square error (MSE) is . This metric accounts for both the variance and the bias in an estimate. However, as mean values of all parameters are indistinguishable from the true values, none of the estimators are biased, and distinctions between are due to variance. The 4PL+C procedure leads to a reduction in MSE that is especially pronounced for *R*_*b*_ (∼0.17 of 4PL) and *R*_*t*_ (∼0.48 of 4PL) and smaller but still statistically significant for *x*_50_ and *H* (∼0.75 of 4PL for both) (Table 1). The change in MSE is larger than the standard deviation across 5 independent sets of curves.

**Table 1:** Comparison of the Error in Parameter Estimates from Simulated CRCs^a^

MSE	4PL+C	4PL	3PLFB	3PLFT	2PL
Low variance					
*R*_*b*_	3.8 × 10^–1^ (1.1 × 10^–2^)	2.3 (4.6 × 10^–2^)		2.3 (4.6 × 10^–2^)	
*R*_*t*_	6.2 × 10^–1^ (1.3 × 10^–2^)	1.3 (2.7 × 10^–2^)	1.3 (2.5 × 10^–2^)		
*x*_50_	4.4 × 10^–4^ (9.1 × 10^–6^)	5.8 × 10^–4^ (8.7 × 10^–6^)	5.0 × 10^–4^ (1.3 × 10^–5^)	6.1 × 10^–4^ (1.0 × 10^–5^)	6.0 × 10^–4^ (1.2 × 10^–5^)
*H*	1.8 × 10^–3^ (2.1 × 10^–5^)	2.4 × 10^–3^ (2.7 × 10^–5^)	2.1 × 10^–3^ (3.1 × 10^–5^)	2.8 × 10^–3^ (3.9 × 10^–5^)	2.2 × 10^–3^ (2.5 × 10^–5^)
High variance					
*R*_*b*_	1.1 (3.5 × 10^–2^)	6.9 (1.4 × 10^–1^)		7.1 (1.4 × 10^–1^)	
*R*_*t*_	1.9 (4.0 × 10^–2^)	3.9 (8.2 × 10^–2^)	3.8 (7.5 × 10^–2^)		
*x*_50_	1.3 × 10^–3^ (2.7 × 10^–5^)	1.7 × 10^–3^ (2.7 × 10^–5^)	1.5 × 10^–3^ (4.0 × 10^–5^)	1.8 × 10^–3^ (3.2 × 10^–5^)	1.8 × 10^–3^ (3.9 × 10^–5^)
*H*	5.6 × 10^–3^ (7.8 × 10^–5^)	7.5 × 10^–3^ (9.6 × 10^–5^)	6.4 × 10^–3^ (1.0 × 10^–4^)	8.8 × 10^–3^ (1.3 × 10^–4^)	6.9 × 10^–3^ (7.5 × 10^–5^)

a Mean and standard deviation (in parentheses) of the MSE of parameter estimates from 5000 simulations across 10 independent sets. Note that the parameters *R*_*b*_ and *R*_*t*_ correspond to normalized data, opposed to parameters *R*_*b*_° and *R*_*t*_° which relate to the original data.

**Figure 2:**
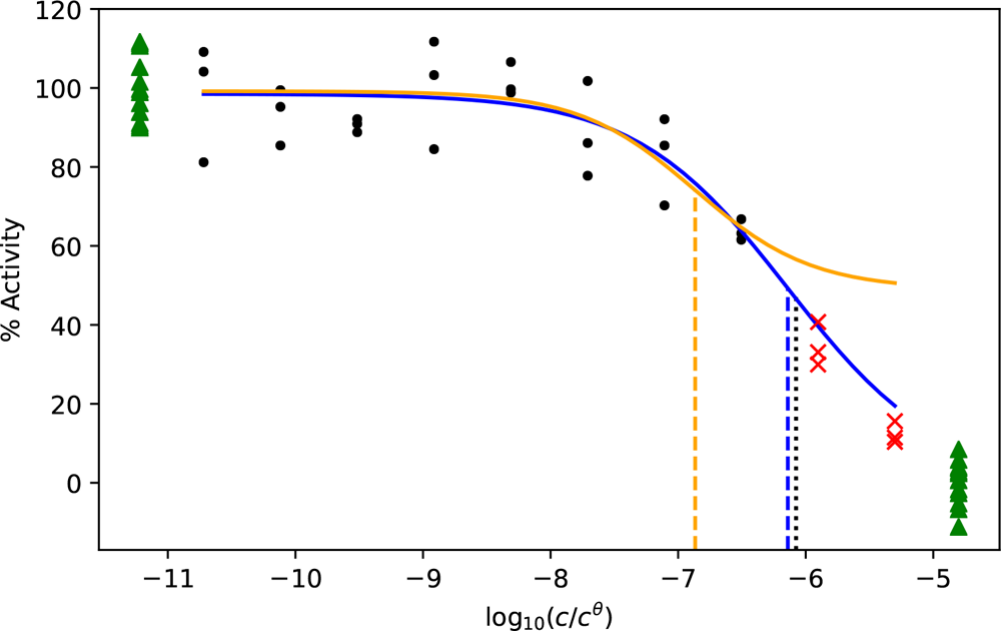
Example fits to incomplete data with (blue line) and without (orange line) controls. Curve data are shown with black circles and control data as green triangles. In the analysis of the incomplete curves, the points represented by red crosses are removed. The *x* position of the control data is not meaningful; data on the left correspond to *x* → –*∞* and on the right correspond to *x* → *∞*. The vertical lines indicate the true *x*_50_ (black dotted) or estimated *x*_50_ from CRC fitting with (blue dashed) or without (orange dashed) controls.

While there are statistically significant benefits to using 4PL+C to analyze complete curves, the improvement is even more evident for incomplete curves. We obtained incomplete curves by removing data corresponding to the two highest concentrations of the simulated completed curves (Figure 2). Removing these data increases the MSE of all parameter estimates (Table 2). While the increase in MSE is approximately 4-fold for the standard 4PL procedure, the increase is subtle for 4PL+C; using the positive control appears to mostly compensate for losing data points at the highest concentrations. Because 3PLFB also uses the controls to set *R*_*b*_, this procedure also compensates for the loss of these data points, and the precision is similar to 4PL+C.

**Table 2:** Comparison of Error in Parameter Estimates from Simulated Incomplete CRCs^a^

MSE	4PL+C	4PL	3PLFB
*R*_*b*_	1.0 × 10^–1^ (3.5 × 10^–3^)	6.6 × 10^1^ (1.0)	
*R*_*t*_	1.9 × 10^0^ (3.9 × 10^–2^)	3.9 (8.2 × 10^–2^)	3.8 (7.9 × 10^–2^)
*x*_50_	1.4 × 10^–3^ (3.1 × 10^–5^)	8.3 × 10^–3^ (1.8 × 10^–4^)	1.5 × 10^–3^ (3.8 × 10^–5^)
*H*	5.8 × 10^–3^ (8.5 × 10^–5^)	2.1 × 10^–2^ (2.8 × 10^–4^)	6.1 × 10^–3^ (9.1 × 10^–5^)

a Mean and standard deviation (in parentheses) of the MSE of parameters estimated from 5000 curves across 10 independent sets.

While it is clear that including control data will improve the estimation of parameters, the extent of improvement will depend on a large number of factors. These factors include the variance of the control and curve data. They also include the set of concentrations at which responses are measured, including the number of points along the curve and whether the curve is complete or incomplete. Hence the aforementioned reduction in the MSE may not be representative of all situations.

Our result that including control data is especially beneficial for the analysis of incomplete curves is consistent with the previous observations. Kappenberg et al.^6^ found that in the “difficult” situation of an incomplete curve, estimation with controls outperforms estimation without controls unless the controls are strongly deviating. Sebaugh^4^ found that fixing *R*_*b*_ or *R*_*t*_ can strongly influence estimates of *x*_50_ and *H* in incomplete curves. As mentioned in the introduction, fixing *R*_*b*_ or *R*_*t*_ privileges the control data by using it (and not the curve data) to set values for these parameters. The 4PL+C approach provides similar benefits for the analysis of incomplete curves without the subjective choice of which parameters to fix and without giving unwarranted privilege to the control data.

### The Precision of Estimates from Repeated Experiments Is Consistent with Trends Observed in Simulations

The COVID Moonshot data include some repeated experiments. The fluorescence assay data include 9 independent CRCs for inhibition of MPro by a compound with database identifier CVD-0002707. The mass spectroscopy assay data include 25 independent CRCs for ebselen. The parameters estimated by applying 4PL versus 4PL+C are shown in Figure S3 of Supporting Information 1. In this figure, we compared 4PL+C to 4PL instead of 3PLFB because the former is more standard and is more widely used. For both data sets, 4PL+C reduces the standard deviation of estimated *R*_*b*_ and *R*_*t*_ compared to 4PL (Table S1 of Supporting Information 1). There was not a statistically significant difference between the standard deviation of pIC_50_ and Hill slope. These results are inconclusive but consistent with the trends observed in the simulated data.

There is a large variation in the estimated Hill slope for ebselen. This is likely because ebselen is a covalent inhibitor. For a covalent inhibitor, the results are dependent on the time between incubation and measurement, which could be highly variable. This variability does not affect the comparison between fitting procedures, as the Hill slope reported for each curve is relatively insensitive to the fitting procedure (Figure S3 of Supporting Information 1).

### 4PL+C Is Reasonably Fast and a Simplification Is Widely Available

Although curve fitting with the 4PL+C procedure is slower than that with 4PL, both are still quite fast. Performing fits for all 1668 fluorescence curves took 6 min and 2 s for 4PL+C but only 18 s for 4PL. For all 238 mass spectrometry curves, the fitting took 36 s for 4PL+C versus 8 s for 4PL. As described in the methods section, the 4PL+C procedure alternates between optimizing the Hill equation parameters **θ** and the estimated variances. In contrast, the 4PL procedure requires optimizing only the Hill equation parameters. Nonetheless, 4PL+C is quite fast, and the additional computing cost required for the curve fitting should not be a barrier to using the procedure.

While existing data analysis software do not, to our knowledge, implement 4PL+C, all nonlinear regression software implement a simplified version of 4PL+C. Some packages like *drc*(12) use model functions that are defined at a concentration of zero. Even in software that do not, Weimer et al.^5^ noted that it is straightforward to include control data by assigning them extreme concentration values such that the model response is a close approximation to the asymptotes. This procedure leads to a simplication of 4PL+C in which the controls are assumed to have the *same* variance as the curve. However, at least for the COVID Moonshot data, we see that this appears to be an unreasonable assumption.

### Variances Differ between the Curve and Controls

**Figure 3:**
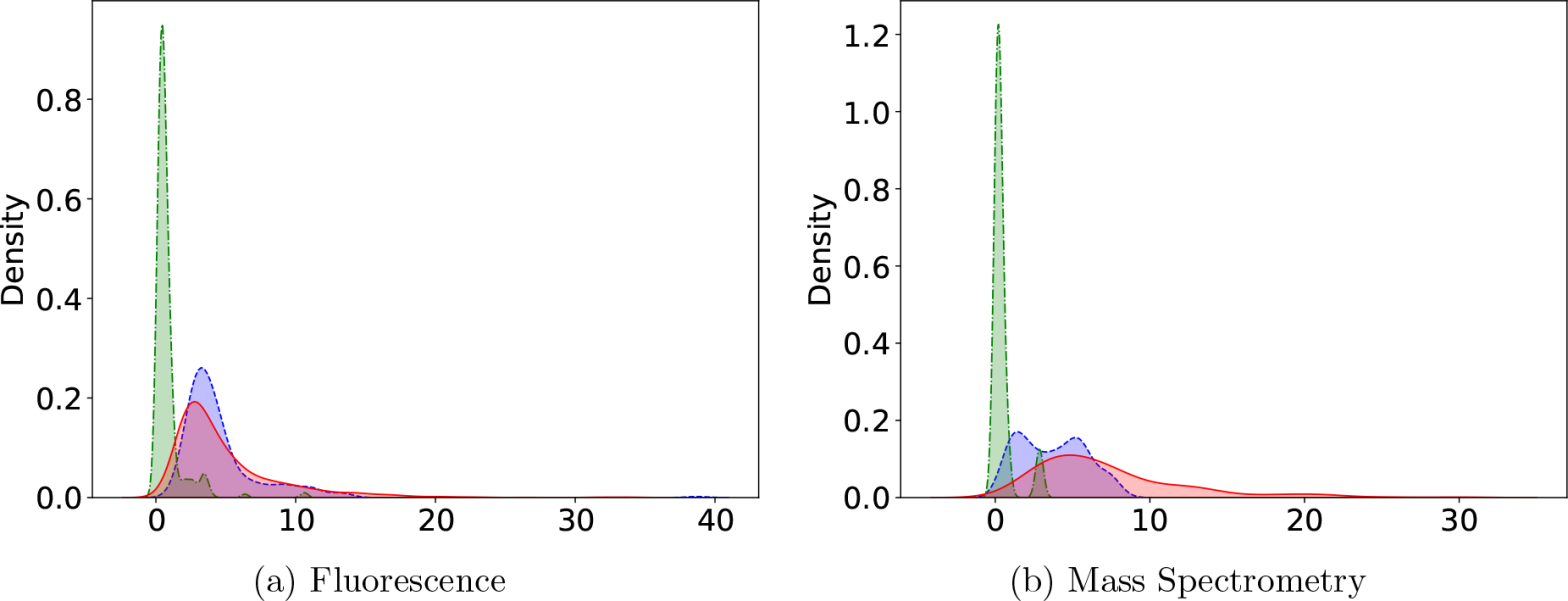
Estimated standard deviations of the controls and of the curves. Histogram of estimated standard deviations of the bottom controls  (green dashed dotted line), the top controls  (blue dashed line), and the curve  (red line) for (a) fluorescence and (b) mass spectroscopy experiments.

In the fluorescence and mass spectroscopy CRC data collected for the COVID Moonshot, the estimated variance of the curve and the controls differ (Figure 3). In both assay types, the bottom control has the least variance. For the fluorescence measurements, the variance of the top controls is comparable to that of the curve. For mass spectroscopy experiments, the variance of the curve is often higher than the top control. In both experiments, there is no correlation between the estimated variance of the top control and the curve (data not shown).

Differences in variance can be explained by experimental settings, which have been described elsewhere.^20^ Bottom controls have buffer but no enzyme and are subject to less variation due to concentration errors that may occur, for example, due to pipetting error or enzyme degradation. In the fluorescence measurements, the top controls and curves have comparable sources of error. In the mass spectroscopy experiments, the top control contains only DMSO (no inhibitor) and does not have error due to the inhibitor concentration.

### The 4PL+C Procedure Reduces the Estimated ASE of Experimental CRCs

**Figure 4:**
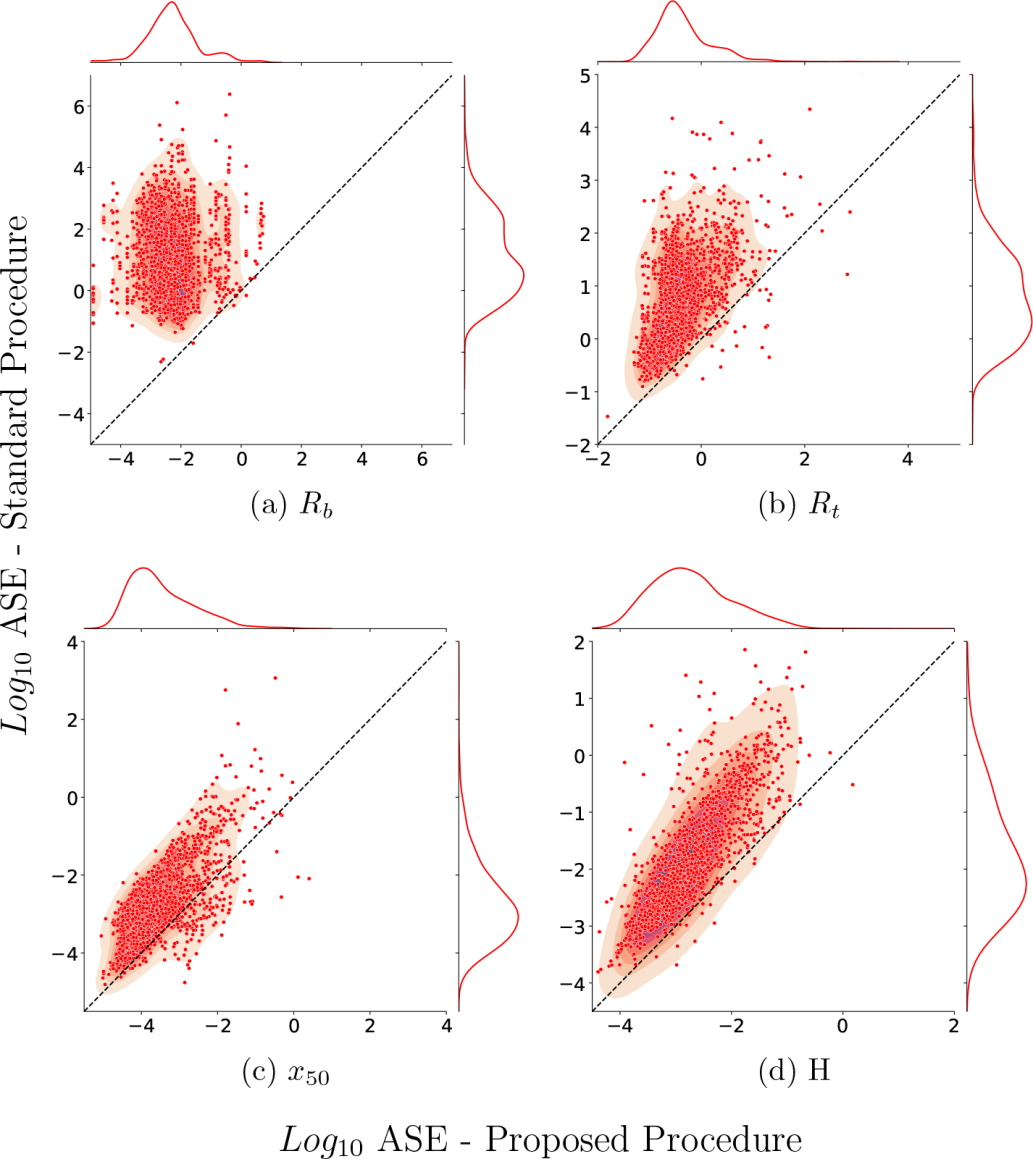
Comparison of ASE estimates for 4PL+C and 4PL analysis of fluorescence CRC from the COVID Moonshot. For each parameter, the fraction of data above the diagonal is *R*_*b*_ (99.69%), *R*_*t*_ (98.16%), *x*_50_ (89.51%), and *H* (98.49%).

**Figure 5:**
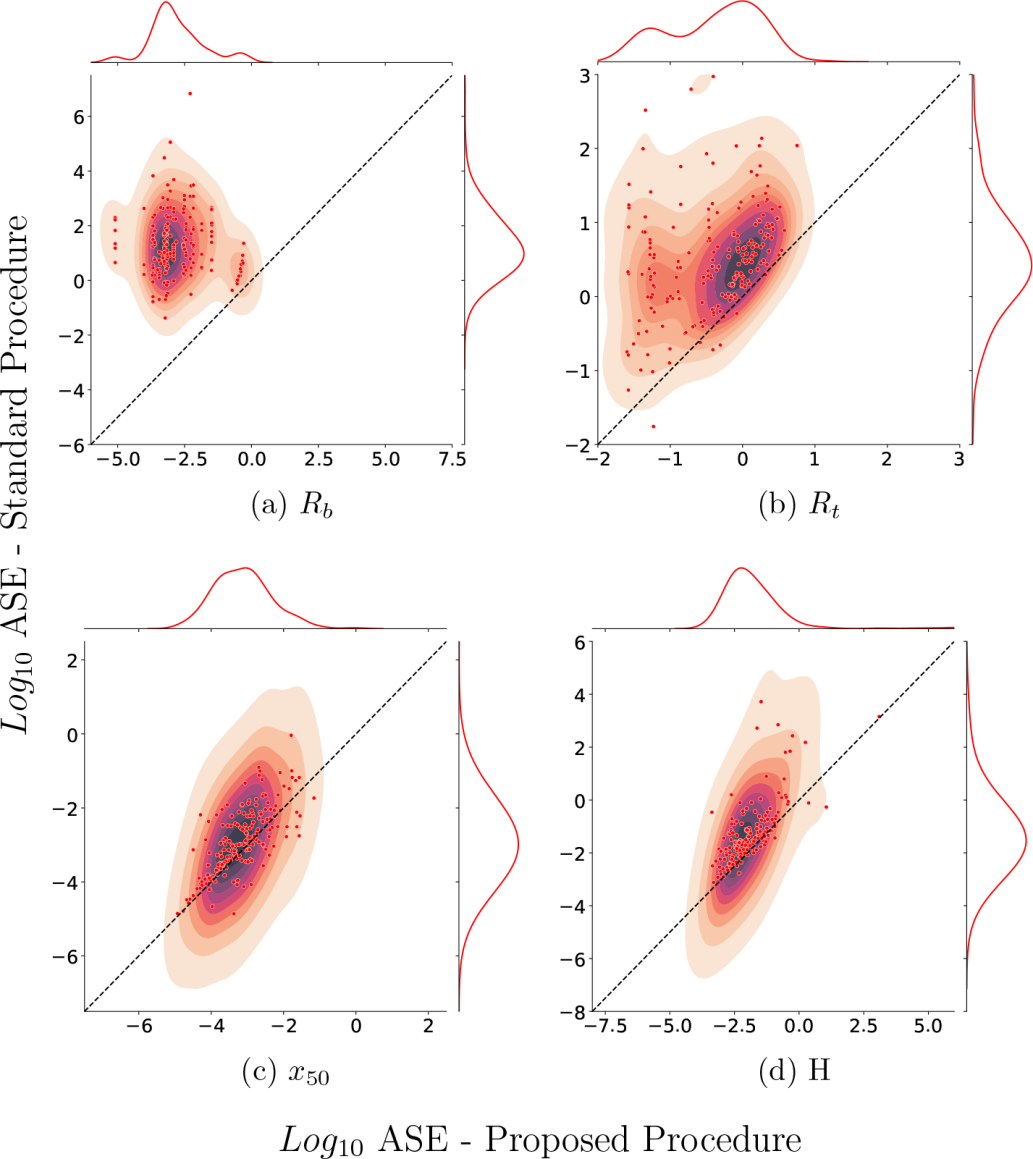
Comparison of ASE estimates for 4PL+C and 4PL analysis of mass spectroscopy CRC from the COVID Moonshot. For each parameter, the fraction of data above the diagonal is *R*_*b*_ (100.00%), *R*_*t*_ (96.57%), *x*_50_ (76.96%), and *H* (88.24%).

For the vast majority of the fluorescence and mass spectroscopy CRC data collected for the COVID Moonshot, 4PL+C yields a smaller asymptotic standard error (ASE) than 4PL. Examples of fitting curves using 4PL+C and 4PL can be found in Supporting Information 2. We fit 1589 of 1668 fluorescence curves and 205 of 238 mass spectrometry curves with a high coefficient of determination, *R*^2^ > 50%, using both procedures. In over 95% of the curves of both types, the ASE of *R*_*t*_ and *R*_*b*_ was lower using 4PL+C than 4PL (Figures 4 and 5). The 4PL+C procedure provides a lower ASE than 4PL in over 75% of the *x*_50_ and *H* estimates. The reduction in ASE suggests that the 4PL+C determines parameters more accurately than 4PL.

### Outlier Detection and Curve Refitting Can Improve the Precision of Parameter Estimates in Both 4PL and 4PL+C

For simulated CRCs, outlier detection and curve refitting did not improve the precision of the parameter estimates. Lenth’s method^21^ detected an outlier in about 40% of curves. However, removing the outliers and refitting had a negligible effect on the MSE of estimated parameters, yielding results identical with those in Table 1. The normally distributed error in the simulated curves does not lead to significant perturbations of parameter estimates.

**Figure 6:**
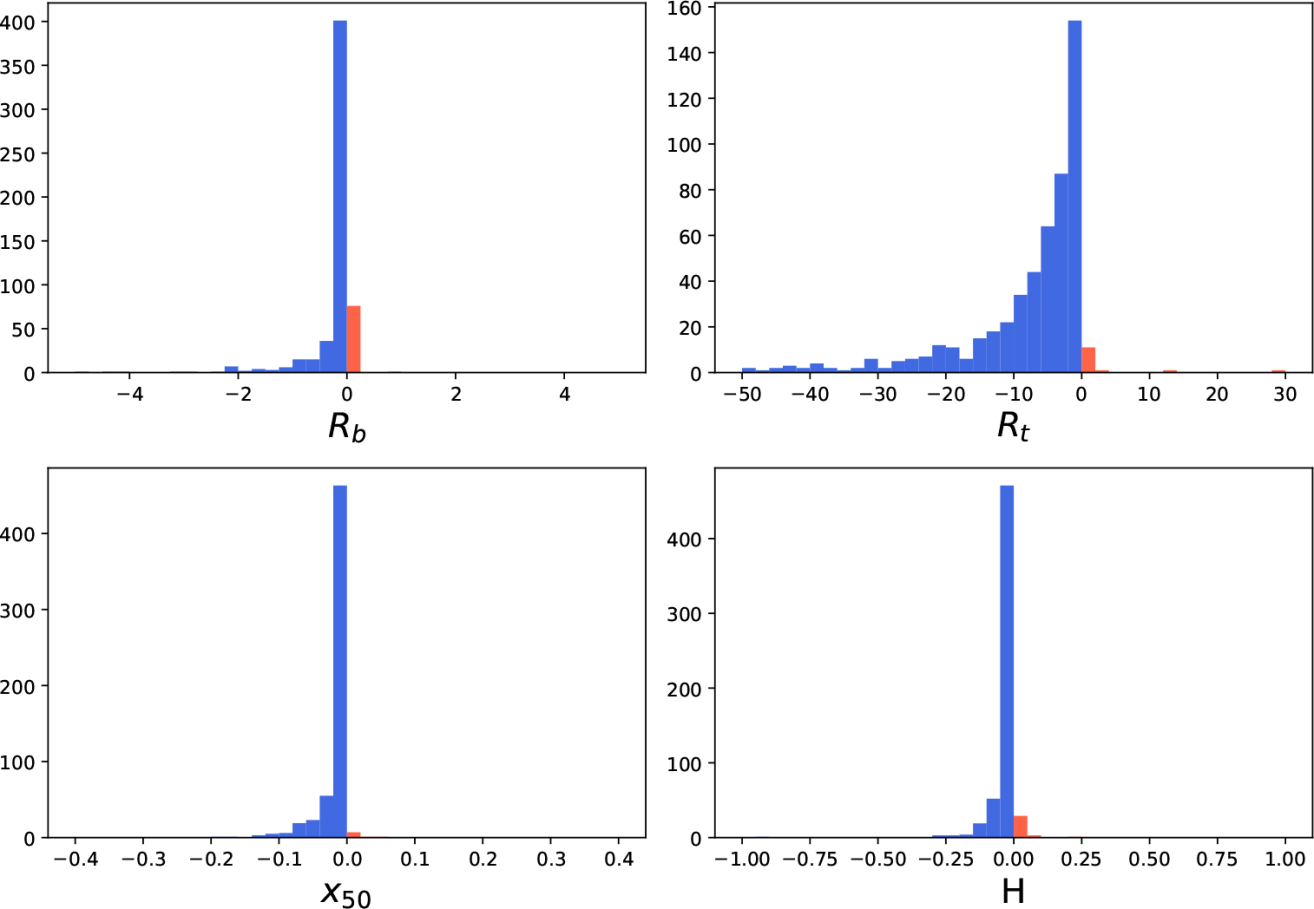
Histogram of the change in ASE after outlier detection and refitting using the 4PL+C procedure for data from the fluorescence assay. In most parameter estimates (*R*_*b*_: 86.86%, *R*_*t*_: 97.44%, *x*_50_: 98.46%, *H*: 94.37%), the ASE is reduced (blue), but in some, the ASE increases (orange).

**Figure 7:**
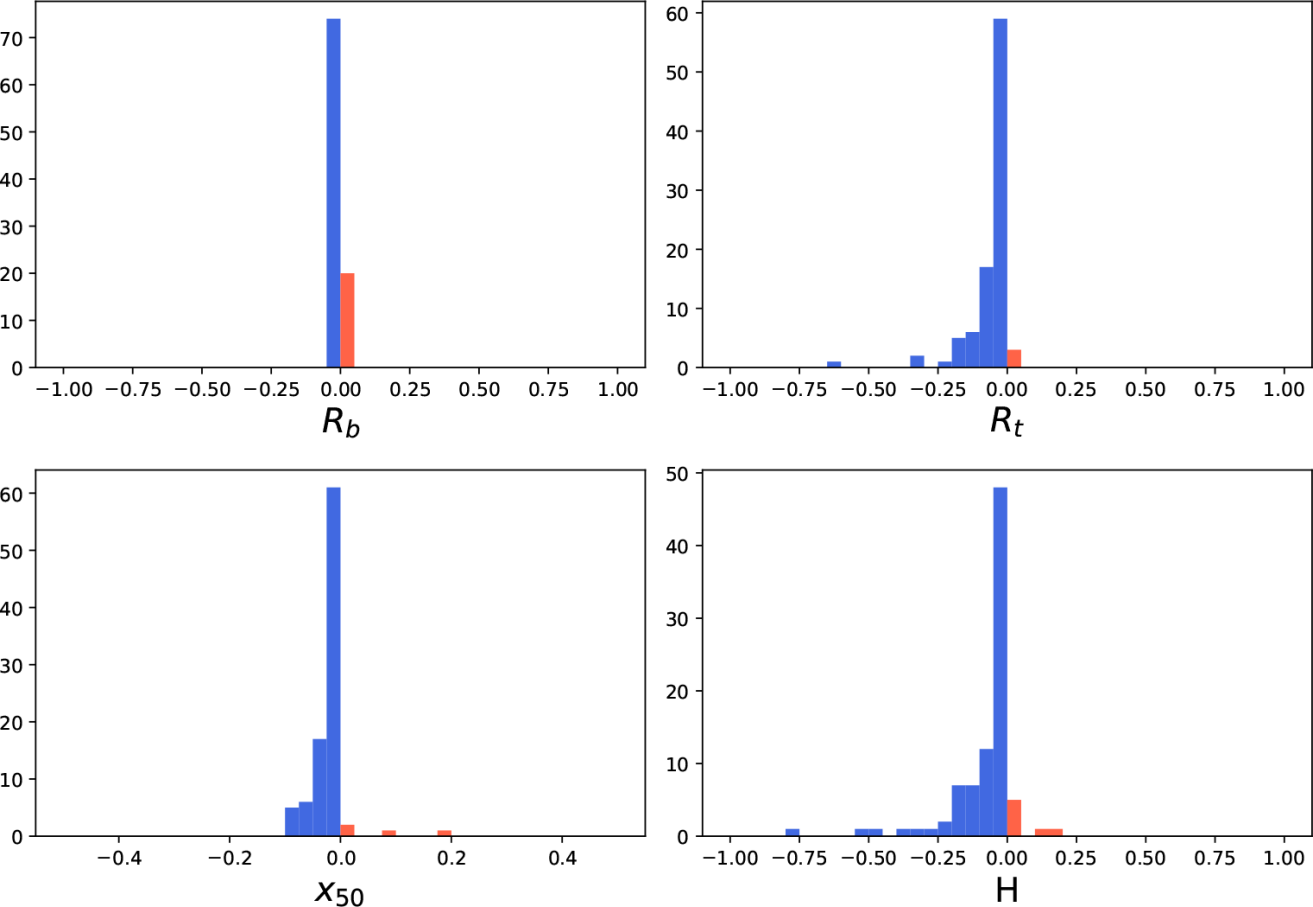
Histogram of the change in ASE after outlier detection and refitting using the 4PL+C procedure for data from the mass spectroscopy assay. In most parameter estimates (*R*_*b*_: 78.72%, *R*_*t*_: 96.81%, *x*_50_: 94.68%, *H*: 89.36%), the ASE is reduced (blue), but in some the ASE increases (orange).

For data from both the fluorescence and mass spectroscopy assays, removing outliers using Lenth’s method^21^ reduces the ASE for the vast majority of estimated parameters. Of the 1668 fluorescence assay curves, 590 had at least one outlier. To show the effect of outlier removal on the precision of each parameter, we plotted a histogram of the difference in the ASE before versus after outlier removal. For the majority of these curves, removing the outlier(s) and refitting the data led to a lower ASE. (Figure 6). Of the 238 mass spectroscopy curves, 94 had at least one outlier. For most of these, removing the outlier(s) and refitting led to lower ASE (Figure 7). Similar results were observed when refitting with the standard procedure (Figure S4 in the Supporting Information 1).

## Conclusions

As demonstrated by analyses of simulation and experimental data, the proposed 4PL+C procedure results in more accurate estimates of Hill equation parameters, including the half-maximal concentration and Hill slope, than the established regression methods. Benefits are especially evident in the analysis of incomplete curves. Moreover, we have shown that an outlier detection and curve refitting leads to a lower ASE of the pIC_50_ and Hill slope for both 4PL+C and 4PL.

The proposed method provides clear benefits without a significant cost. No more data need to be collected than is required for standard statistical approaches. Implementation is only minimally more complex. Computational expense increases are not prohibitive. While most curve fitting software can perform a variant of 4PL+C in which all control and curve data are assumed to have the same variance, this appears to be, at least with the COVID Moonshot data, a bad assumption. Thus, we recommend that the described 4PL+C procedure, with different variances for the curve and top and bottom controls, be implemented by data analysis software developers. We recommend that it be applied by scientists as a new standard, replacing 4PL.

## Experimental Section

### Statistical Approaches

#### Maximum Likelihood Estimation without Control Data

Our procedure is based on the maximum likelihood estimation of parameters that fit the Hill equation to CRC data. We use  to denote the experimental data on the curve . Here *x*_*i*_’s are base 10 logarithms of concentrations of the compound where response measurements are recorded, and we assume that there are *m* unique *x*_*i*_ values. At each *x*_*i*_, there are *n*_*i*_ repeated measurements of response *r*_*i*,*j*_ for *j* = 1, ..., *n*_*i*_. Therefore, there are *N*_*c*_=∑_*i* = 1_^*m*^*n*_*i*_ observations that are part of the concentration–response curve. The most common practice is to use the same number of replicates at each *x*_*i*_, such that *n*_*i*_ = *n* for *i* = 1, ..., *m* and *N*_*c*_ = *m* × *n*.

To obtain the likelihood function of the CRC, we assume that the data follow the classical Hill equation in eq 1 with an additive measurement error:
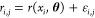
2The measurement error ϵ_*i*,*j*_ independently and identically follows the normal distribution  with constant variance σ_*c*_^2^. Unknown parameters include **θ** = (*R*_*t*_, *R*_*b*_, *x*_50_, *H*) in the Hill equation and the nuisance parameter σ_*c*_^2^. Based on these model assumptions, the log-likelihood of the CRC data is
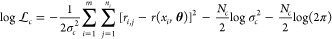
3The maximum likelihood estimator (MLE) is the solution of the following maximization problem:
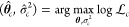
4

Note that for the parameters that maximize the likelihood, the gradient of the log likelihood with respect to  is zero. This condition may be expressed in two equations:  and , where . Due to the structure of , the two equations can be solved separately. Since the Hill equation is nonlinear in **θ**, it is suitable to use a nonlinear equation solver to solve the first equation; this is the typical nonlinear regression approach. Thus, the MLE of  is . However, the MLE of σ_*c*_^2^ is actually biased. We instead use an unbiased estimator of σ_*c*_^2^:
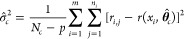
where *p* = 4 is the number of parameters in **θ**.

#### Maximum Likelihood Estimation Using Control Data

In many cases, it is reasonable to use control data as measurements of extreme responses. Consider an enzyme inhibition assay. For a negative control without any inhibitor, because it is the base 10 logarithm of the concentration, *x* is undefined, and the data technically are not part of the curve. However, it is reasonable to treat the negative control data as corresponding to an extremely low concentration such that *x* → –*∞*. For a positive control with substrate(s) and cofactor(s) but no enzyme, the response should be the same as if the enzyme was present but fully inhibited by an extremely high concentration of inhibitor, such that *x* → *∞*.

We obtain the likelihood of the control data based on the assumptions that they correspond to extreme responses and that measurement error has a similar structure to the curve. The bottom control data  include the observations *r*_*b*,*j*_ for *j* = 1, ..., *N*_*b*_ and are considered as measurements in which *x* is extremely large, such that *x* → *∞*. In this limit, the Hill equation yields *R*_*b*_ and measured responses are *r*_*b*,*j*_ = *R*_*b*_ + ϵ_*b*,*j*_, where ϵ_*b*,*j*_’s are the measurement errors. Because the controls may be measurements of solutions containing substances different from the curve, we cautiously assume that the measurement errors follow different normal distributions for the control and curve data. Specifically, ϵ_*b*,*j*_’s independently and identically follow the normal distribution , where σ_*b*_^2^ is a constant, the variance of the measurement error. Like σ_*c*_^2^, σ_*b*_^2^ is an unknown nuisance parameter. At the other extreme, the top control data  include the observations *r*_*t*,*j*_ for *j* = 1, ..., *N*_*t*_ and correspond to *x* → –*∞*. Based on the Hill equation in this limit, *r*_*t*,*j*_ = *R*_*t*_ + ϵ_*t*,*j*_, where  and are independently and identically distributed. Based on these assumptions, the log-likelihood of a bottom controls is,
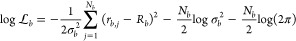
5and of the top controls is
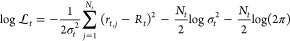
6

The log-likelihood of curve *and* control data is simply the sum of the log-likelihood of each group of data:
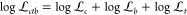
7The MLE of the parameters based on both the curve and control data is the solution of
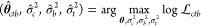
8The two estimators  and  are different as the former is only based on the data set ***D***_*c*_, whereas the latter is based on ***D***_*c*_, ***D***_*t*_, and ***D***_*b*_. Moreover, the estimators maximize different likelihood functions (eq 4 versus eq 8).

Solving eq 8 is slightly more complicated than solving eq 4. Maximizing  is equivalent to minimizing *R*(**θ**,σ_*c*_^2^,σ_*t*_^2^,σ_*b*_^2^), where
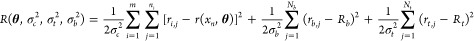
9At the minimal *R*(**θ**,σ_*c*_^2^,σ_*t*_^2^,σ_*b*_^2^), the gradients are zero, such that , , and . Compared to the MLE without control data, the equations for **θ** and (σ_*c*_^2^,σ_*t*_^2^,σ_*b*_^2^) are more intertwined. Therefore, we iteratively solve these equations in terms of **θ** for fixed (σ_*c*_^2^,σ_*t*_^2^,σ_*b*_^2^) values and then update (σ_*c*_^2^,σ_*t*_^2^) values using the latest solution of **θ**. Iteration are stopped when some convergence conditions are achieved: when the changes in *R* function values and parameter values are less than a prespecified tolerance value. Denoting  as the optimal solution for **θ**, the estimates for the three variances are
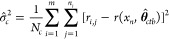
10
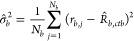
11
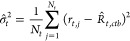
12Compared to the fitting without control data, it is more complicated to adjust the estimates to correct the bias for . For larger sample sizes, such as in this study, the bias is not substantial.

#### Inference of Asymptotic Standard Error

Next, we discuss the asymptotic standard error of the two MLEs. In general, the asymptotic distribution of an MLE **θ̂** is normal with zero mean and covariance given by the inverse of the Fisher information matrix, i.e.,
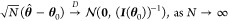
13where *N* and **θ** are the generic notation for sample size and parameters, and **θ**_0_ is the vector of the true parameter values. The matrix ***I***(**θ**) is the Fisher information matrix evaluated at **θ** and defined as
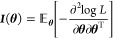
Since **θ**_0_ is unknown, *I*(**θ**_0_) is usually approximated by the sample mean,
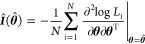
14where *L*_*i*_ is the likelihood of the *i*th observation. Consequently, the asymptotic covariance of **θ̂** is
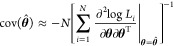
15The ASE of each parameter is simply the diagonal of the asymptotic covariance matrix. Therefore, a confidence interval of (1 – α) × 100% is given by , where *z*_α/2_ is the number of standard deviations (z-score) for the given value of α/2 such that the probability of a variable being contained in the interval is Pr(*Z* ≤ *z*_α/2_) = 1 – α/2.

To infer the ASE for the MLE based on data excluding controls (eq 4), we used eq 3 in the general ASE expression (eq 15). This leads to,
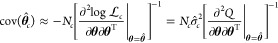
16The gradient and Hessian of *Q* are given by
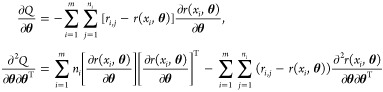
Detailed formulas for gradient and Hessian of *r*(*x*_*i*_, **θ**) are given in Appendix S1 of Supporting Information 1. To compute , we can plug in either the MLE or unbiased estimator for .

To infer the ASE for the MLE based on data including controls (eq 8), we use eq 7 in the general ASE expression. In this case, the covariance of **θ**_*ctb*_ is approximately,
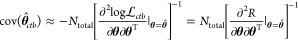
17where *N*_total_ = *N*_*c*_ + *N*_*t*_ + *N*_*b*_ is the total number of observations from all the data sets. The gradient and Hessian of R are given in Appendix S2 of Supporting Information 1. We plug in the estimates eqs 10, 11, and 12 to compute .

#### Normalization

Thus, far in the Statistical Approaches, we have not discussed the effect of normalization. It is a common practice in the analysis of the CRC experiments to normalize the data prior to fitting the Hill equation model. Normalization helps “accommodate minor changes to assay protocols to ensure robustness”, a key goal of the AGM.^2^ Minor changes in the assay protocols could include variations in the amount of time between the initiation of the enzymatic activity and measurement of the product concentration. Here we address the question of how normalization affects the MLE estimators  and .

Normalization is a linear transformation of the original data *r*_*i*,*j*_° for *j* = 1, ..., *n*_*i*_ and *i* = 1, ..., *m*,
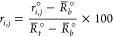
18to yield a percentage response. As explained in the chapter “Data Standardization for Results Management” in the AGM, in most assays, the bottom and top of the curve are based on the controls. Usually,  and  are sample means of the two sides’ control data. It is also possible to use their medians, which are less sensitive to outliers.

Based on the same model as in eq 2, the original data are denoted by *r*_*i*,*j*_° and
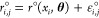
19where *r*^◦^(*x*, **θ**) is,
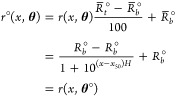
where  and . Compared to the normalized measurement error, the original measurement error ϵ_*i*,*j*_° is scaled, . Hence, the variance of ϵ_*i*,*j*_° is . Thus, if we use the original data {*r*_*i*,*j*_°,*x*_*i*_} for *j* = 1, ..., *n*_*i*_ and *i* = 1, ..., *m*, the corresponding parameters are **θ**°=(*R*_*b*_°, *R*_*t*_°, *x*_50_, *H*) with *R*_*b*_° and *R*_*t*_° defined above and *x*_50_ and *H* remaining the same as in the normalized formulation.

The procedure for obtaining the MLE is exactly the same as for normalized data (see the sections entitled Maximum Likelihood Estimation without Control Data and Maximum Likelihood Estimation Using Control Data) except the data are replaced with original data and the parameters are changed to **θ**^◦^. The log-likelihood of the original data (excluding control data) is
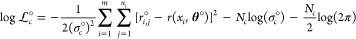
20It is easy to see that the MLE for **θ**^◦^, denoted by , has the same relationship with , as **θ**′ with **θ**, and the MLE for *x*_50_ and *H* remains the same whether the data are normalized or not.

If the control data are included in the estimation, would the normalization affect the estimator ? No, normalization does not affect the estimator  either. Note the relationships:
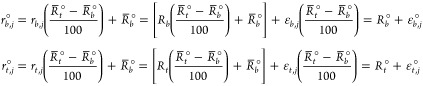
where  and , , and *R*_*b*_° and *R*_*t*_° are previously defined. Therefore, the models for the normalized control data follow the same model as the unnormalized ones as long as the parameters are transformed accordingly. Using the same logic as for , we can directly conclude that the MLE  using the original data have the same estimates for *x*_50_ and *H* as using the normalized data. For *R*_*b*_ and *R*_*t*_, using either one of the two approaches, it is always the case that  and .

### Data Analysis

#### Data

We analyzed simulated and experimental data. Simulations are advantageous because we know the true values and can repeat calculations many times. For experimental data, even without true values and as many repetitions, it is helpful to compare the estimated ASE for different statistical analyses. We analyzed experimental data from the COVID Moonshot project.^20^ The COVID Moonshot was a worldwide collaboration to develop an open-source antiviral that treats COVID-19 by targeting the SARS-CoV-2 main protease. We analyzed CRC in which protease activity was measured by fluorescence (1668 curves) and mass spectrometry (238 curves). These data sets can be found in an Excel spreadsheet as Supporting Information 2.

CRC data were simulated by adding random Gaussian noise to the classical Hill equation. Parameters for the Hill equation were selected to be comparable to mass spectrometry measurements of enzymatic inhibition assays from the COVID Moonshot:^20^*R*_*b*_° and *R*_*t*_° were set at 0.227 and 54.074, while *H* was fixed at 1 and *x*_50_ at −6.0759. For the simulated curves, 10 concentrations were used, with the highest concentration at 100 μM and the others diluted by a factor of 5 each. Random Gaussian noise with mean 0 and variance  was added. The number of replicates for each concentration on the curve was *n* = 4 and the number of observations of each control was *N*_*b*_ = *N*_*t*_ = 16. Simulations were performed in low-variance (, ) and high-variance (, ) conditions. In each condition, 10 sets of 5000 curves were simulated. Incomplete curves were obtained by removing data corresponding to the two highest concentrations from simulations in the high-variance condition.^4^

#### Curve Fitting

All of the statistical approaches, 4PL+C, 4PL, 3PLFB, 3PLFT, and 2PL, were implemented in Python. The code is freely available at https://github.com/vanngocthuyla/crc/tree/main/nls_crc. Maximization of the log likelihood was performed using functions curve_fit and minimize from scipy.optimize.^22^ Initial values of *R*_*b*_ and *R*_*t*_ were set at the minimum and maximum values of the percentage activity. During the fitting procedure, these parameters were restricted to their initial values, plus/minus a quarter of the data range. For *x*_50_, the initial value was set to be observed concentration with the response closest to the mean of the minimum and maximum response. The initial Hill slope was set to 1. *x*_50_ and *H* were restricted to the ranges from −50 to 0 and from 0 to 50, respectively. Optimization was performed until the absolute difference in the log likelihood was less than a tolerance of 1 × 10^4^. If this tolerance was not achieved before 100 iterations, no fitted values were reported.

Fitting was performed on a computer cluster with the following key specifications: a dual AMD Opteron 6376 processor with a clock speed of 2.30 GHz and 16MB cache, 32 cores, and 64 GB RAM. The job required one core and 2 GB of RAM.

#### Outlier Detection and Curve Refitting

In addition to analyses without preprocessing, we also performed some analyses of experimental data with automated outlier detection based on Lenth’s method.^21^ Starting with the residual between the observed data and model values from the initial fit, , *s*_0_ = 1.5*median*|ϵ_*i*,*j*_|. The pseudostandard error (PSE) is defined as
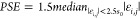
21For every data point, the coefficient of variance is the ratio of the residual, ϵ_*i*,*j*_, over the PSE
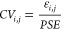
22A data point with CV larger than 1.5 times the interquartile range—the distance between 25^th^ (Q1) and 75^th^ (Q3) quantiles of all *CV*_*i*,*j*_—is considered to be an outlier and is removed.^23^ If a curve has at least one outlier, the outliers are removed, and the curve is refit.

## Supporting Information Available

Appendices for the gradients and Hessians of functions reference in the main text, histograms of parameter estimates from simulated CRCs, parameter estimates and precision thereof from repeated experiments, and a histogram summarizing the effects of outlier detection in the ASE of the 4PL procedure (PDF)
CRCs from the COVID Moonshot and example fits with and without control data (XLSX)

## Abbreviations Used

2PL: two parameter logistic
3PLFB: three-parameter logistic fixed bottom
3PLFT: three-parameter logistic fixed top
4PL: four parameter logistic
4PL+C: four parameter logistic with controls
AGM: Assay Guidance Manual
ASE: asymptotic standard error
COVID: coronavirus disease
CRC: concentration response curve
MLE: maximum likelihood estimator
MSE: mean square error
SARS-CoV-2: severe acute respiratory syndrome coronavirus 2
