# Supplementary material for: Inclusion of Control Data in Fits to Concentration–Response Curves Improves Estimates of Half-Maximal Concentrations
Source: J Med Chem. 2023 Sep 12;66(18):12751–61. doi: 10.1021/acs.jmedchem.3c00107 (PMC10544339; doi:10.1021/acs.jmedchem.3c00107)
Supplement: Supplementary file 1 — jm3c00107_si_001.pdf [file jm3c00107_si_001.pdf]

Supporting Information for "Inclusion of control data in fits to concentration-response curves improves estimates of half-maximal concentrations"

Van Ngoc Thuy La,[†] Stanley Nicholson,[‡] Amna Haneef,[†] Lulu Kang,[‡] and David D. L. Minh[*,¶]

†*Department of Biology, Illinois Institute of Technology, Chicago, IL 60616, USA*

‡*Department of Applied Mathematics, Illinois Institute of Technology, Chicago, IL 60616, USA*

¶*Department of Chemistry, Illinois Institute of Technology, Chicago, IL 60616, USA*

E-mail: dminh@iit.edu

Contents

Appendix S1: Gradient and Hessian of $r(x, \boldsymbol{\theta})$ S2
Appendix S2: Gradient and Hessian of R S3
Table S1: Comparison of precision of estimates from repeated experiments between 4PL+C, 4PL, and 3PLFB procedures S4
Figure S1: Histograms of estimates from simulated CRCs with low variance S5
Figure S2: Histograms of estimates from simulated CRCs with high variance S6
Figure S3: Parameters estimated by 4PL and 4PL+C in repeated experiments S7
Figure S4: Histogram of changes in the ASE after outlier detection and refitting using the 4PL procedure S8

Appendix S1: Gradient and Hessian of $r(x, \boldsymbol{\theta})$

The gradient of $r(x, \boldsymbol{\theta})$ is,

$$\begin{aligned}
\frac{\partial r(x,\boldsymbol{\theta})}{\partial R_b} &= 1 - (1 + 10^{Hx - Hx_{50}})^{-1}, \\
\frac{\partial r(x,\boldsymbol{\theta})}{\partial R_t} &= (1 + 10^{Hx - Hx_{50}})^{-1}, \\
\frac{\partial r(x,\boldsymbol{\theta})}{\partial x_{50}} &= \frac{\ln(10)(R_t - R_b)H10^{Hx - Hx_{50}}}{(1 + 10^{Hx - Hx_{50}})^2}, \\
\frac{\partial r(x,\boldsymbol{\theta})}{\partial H} &= -\frac{\ln(10)(R_t - R_b)10^{Hx - Hx_{50}}(x - x_{50})}{(1 + 10^{Hx - Hx_{50}})^2}.
\end{aligned}$$

The Hessian is,

$$\begin{aligned}
\frac{\partial^2 r(x,\boldsymbol{\theta})}{\partial R_b^2} &= \frac{\partial^2 r(x,\boldsymbol{\theta})}{\partial R_b \partial R_t} = 0, \quad \frac{\partial^2 r(x,\boldsymbol{\theta})}{\partial R_t^2} = \frac{\partial^2 r(x,\boldsymbol{\theta})}{\partial R_t \partial R_b} = 0 \\
\frac{\partial^2 r(x,\boldsymbol{\theta})}{\partial x_{50}^2} &= [\ln(10)]^2 (R_b - R_t) H^2 \frac{10^{Hx_{50} - Hx}(10^{Hx_{50} - Hx} - 1)}{(1 + 10^{Hx_{50} - Hx})^3}, \\
\frac{\partial^2 r(x,\boldsymbol{\theta})}{\partial H^2} &= [\ln(10)]^2 (R_b - R_t)(x_{50} - x)^2 \frac{10^{Hx_{50} - Hx}(10^{Hx_{50} - Hx} - 1)}{(1 + 10^{Hx_{50} - Hx})^3}, \\
\frac{\partial^2 r(x,\boldsymbol{\theta})}{\partial R_b \partial x_{50}} &= \frac{\partial^2 r(x,\boldsymbol{\theta})}{\partial x_{50} \partial R_b} = -\frac{\ln(10)10^{Hx_{50} - Hx}H}{(1 + 10^{Hx_{50} - Hx})^2} \\
\frac{\partial^2 r(x,\boldsymbol{\theta})}{\partial R_b \partial H} &= \frac{\partial^2 r(x,\boldsymbol{\theta})}{\partial H \partial R_b} = -\frac{\ln(10)10^{Hx_{50} - Hx}(x_{50} - x)}{(1 + 10^{Hx_{50} - Hx})^2} \\
\frac{\partial^2 r(x,\boldsymbol{\theta})}{\partial R_t \partial x_{50}} &= \frac{\partial^2 r(x,\boldsymbol{\theta})}{\partial x_{50} \partial R_t} = \frac{\ln(10)10^{Hx_{50} - Hx}H}{(1 + 10^{Hx_{50} - Hx})^2} \\
\frac{\partial^2 r(x,\boldsymbol{\theta})}{\partial R_t \partial H} &= \frac{\partial^2 r(x,\boldsymbol{\theta})}{\partial H \partial R_t} = \frac{\ln(10)10^{Hx_{50} - Hx}(x_{50} - x)}{(1 + 10^{Hx_{50} - Hx})^2} \\
\frac{\partial^2 r(x,\boldsymbol{\theta})}{\partial x_{50} \partial H} &= \frac{\partial^2 r(x,\boldsymbol{\theta})}{\partial H \partial x_{50}}
\end{aligned}$$

$$= -\frac{[\ln(10)](R_b - R_t)10^{Hx_{50} - Hx}}{(1 + 10^{Hx_{50} - Hx})^3} \left[(Hx_{50} - Hx)\ln(100) - (10^{Hx_{50} - Hx} + 1)((Hx_{50} - Hx)\ln(10) - 1) \right].$$

Appendix S2: Gradient and Hessian of R

The function R is defined as,

$$R = \frac{1}{\sigma_c^2} Q + \frac{1}{2\sigma_b^2} \sum_{j=1}^{N_b} (r_{b,j} - R_b)^2 + \frac{1}{2\sigma_t^2} \sum_{j=1}^{N_t} (r_{t,j} - R_t)^2.$$

Therefore, the gradient and Hessian of R with respect to $\boldsymbol{\theta}$ are

$$\begin{aligned}
\frac{\partial R}{\partial \boldsymbol{\theta}} &= \frac{1}{\sigma_c^2} \frac{\partial Q}{\partial \boldsymbol{\theta}} + [-\frac{1}{\sigma_b^2} \sum_{j=1}^{N_b} (r_{b,j} - R_b), 0, 0, 0]^\top + [0, -\frac{1}{\sigma_t^2} \sum_{j=1}^{N_t} (r_{t,j} - R_t), 0, 0]^\top \\
&= -\frac{1}{\sigma_c^2} \sum_{n,m} (r_{n,m} - r(x_{n,m}, \boldsymbol{\theta})) \frac{\partial r(x_{n,m}, \boldsymbol{\theta})}{\partial \boldsymbol{\theta}} - [\frac{1}{\sigma_b^2} \sum_{j=1}^{N_b} (r_{b,j} - R_b), 0, 0, 0]^\top - [0, \frac{1}{\sigma_t^2} \sum_{j=1}^{N_t} (r_{t,j} - R_t), 0, 0]^\top, \\
\boldsymbol{H}_R &= \frac{1}{\sigma_c^2} \boldsymbol{H}_Q + \frac{N_b}{\sigma_b^2} \boldsymbol{E}_1 + \frac{N_t}{\sigma_t^2} \boldsymbol{E}_2,
\end{aligned}$$

where $\boldsymbol{E}_i$ is a matrix with all entries equal to zero, except $\boldsymbol{E}_i[i, i] = 1$ for $i = 1, 2$.

Table S1: Comparison of precision of estimates from repeated experiments between 4PL+C, 4PL, and 3PLFB procedures

A. Fitting of repeated experiments from the fluorescence dataset

The sample mean and standard deviation (in parentheses) are shown

	4PL+C	4PL	3PLFB
R_b	-1.5E-2 (2.2E-2)	7.6E-2 (2.6E+0)	
R_t	1.1E+2 (1.5E+1)	1.2E+2 (5.1E+1)	1.2E+2 (5.1E+1)
pIC_{50}	5.2E+0 (2.3E-1)	5.2E+0 (2.8E-1)	5.2E+0 (2.8E-1)
H	1.2E+0 (4.8E-1)	1.1E+0 (1.8E-1)	1.1E+0 (2.2E-1)

B. Fitting of repeated experiments from the mass spectrometry dataset

The sample mean and standard deviation (in parentheses) are shown

	4PL+C	4PL	3PLFB
R_b	-4.0E-2 (1.9E-1)	-6.9E-1 (2.2E+0)	
R_t	1.0E+2 (2.4E+0)	1.0E+2 (5.9E+0)	1.0E+2 (6.6E+0)
pIC_{50}	6.9E+0 (1.9E-1)	6.9E+0 (2.2E-1)	6.9E+0 (2.2E-1)
H	2.0E+0 (1.1E+0)	2.0E+0 (1.1E+0)	2.0E+0 (1.1E+0)

Figure S1: Histograms of estimates from simulated CRCs with low variance

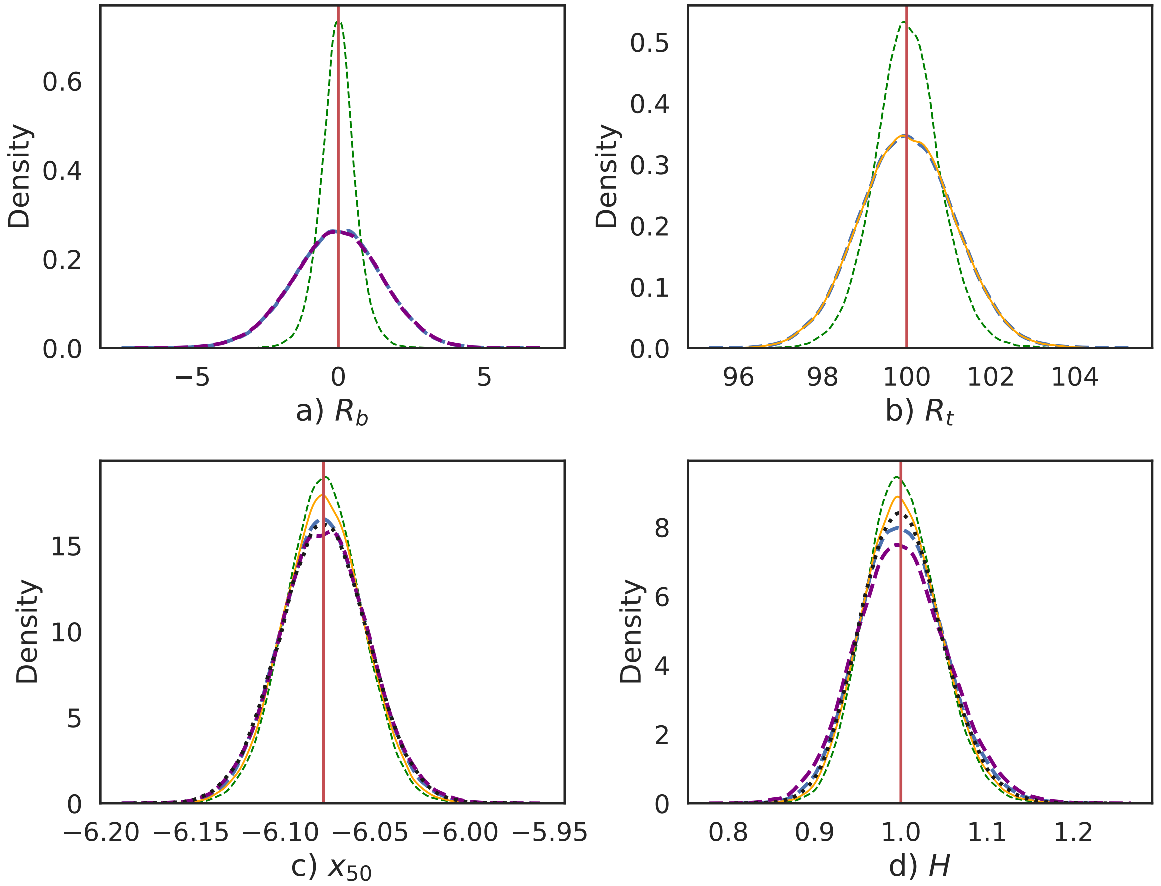

Normalized histogram of estimated parameters from the 4PL+C (green dashed line), 4PL (blue dashed line), 3PLFB (orange solid line), 3PLFT (purple dashed line), and 2PL (black dotted line) procedures. R_b and R_t are normalized. The red solid line shows the true values.

Figure S2: Histograms of estimates from simulated CRCs with high variance

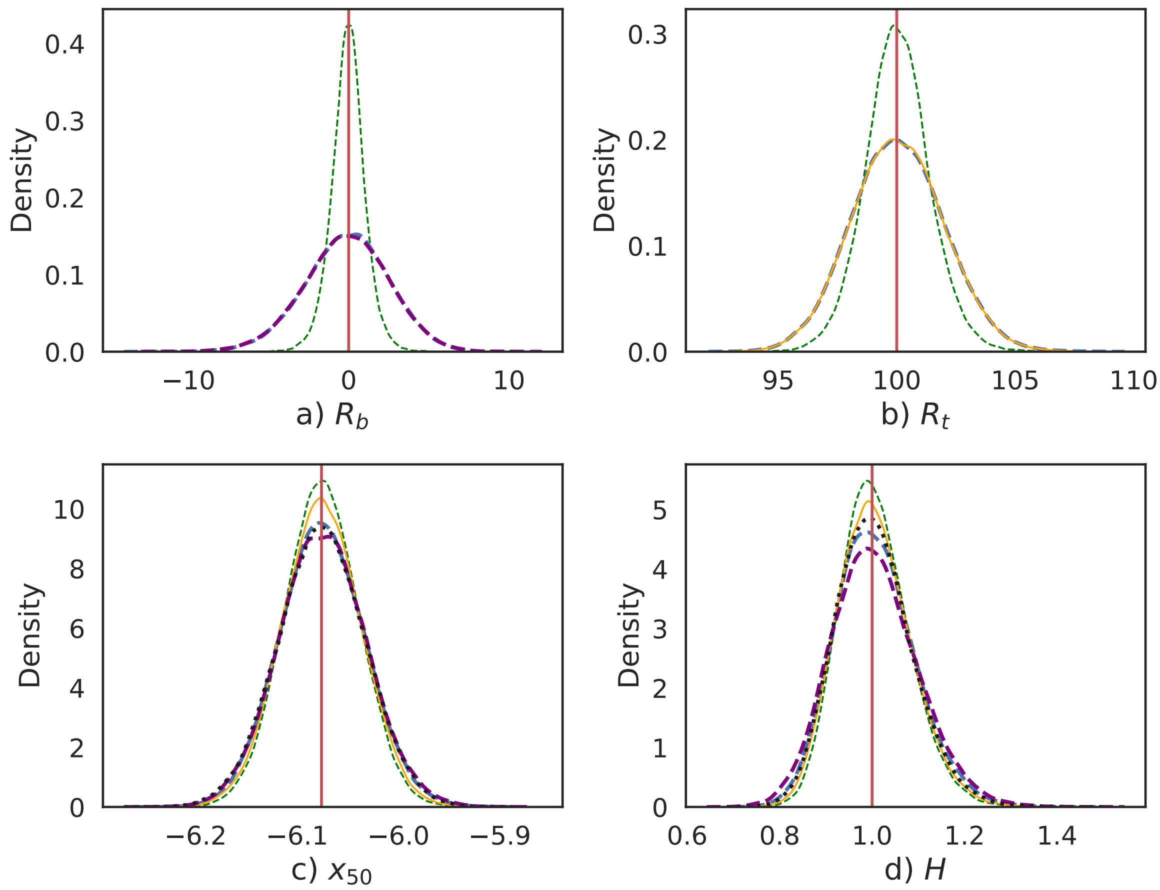

Normalized histogram of estimated parameters from the 4PL+C (green dashed line), 4PL (blue dashed line), 3PLFB (orange solid line), 3PLFT (purple dashed line), and 2PL (black dotted line) procedures. R_b and R_t are normalized. The red solid line shows the true values.

Figure S3: Parameters estimated by 4PL and 4PL+C in repeated experiments

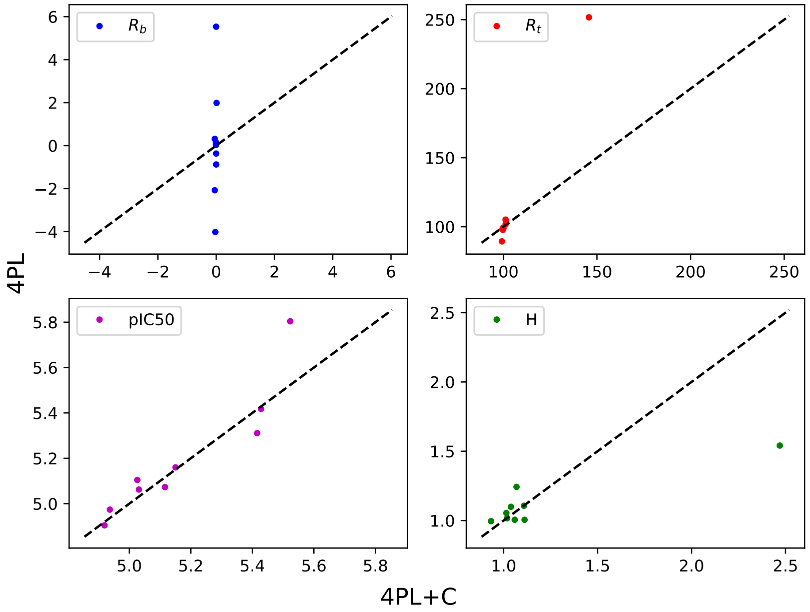

A. Parameters estimated for CVD-0002707, in which the product concentration was measured by fluorescence

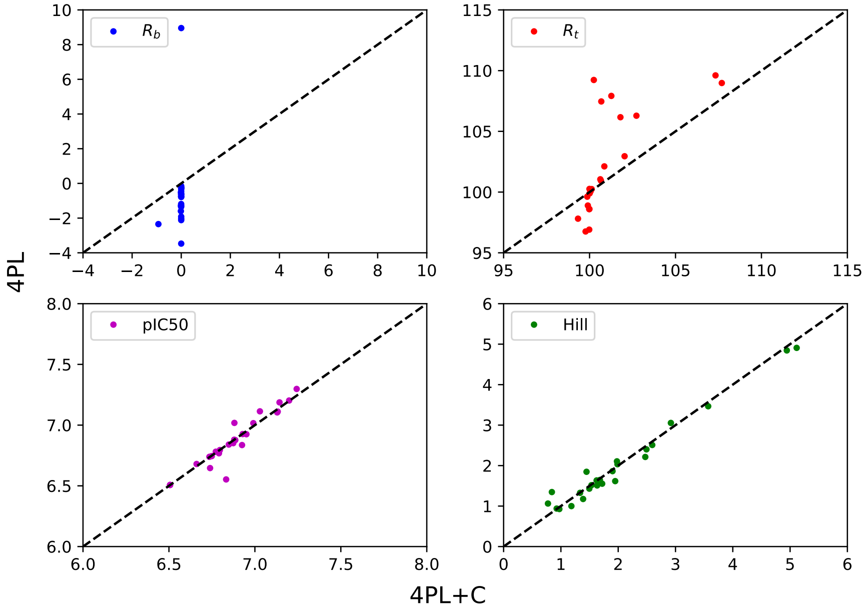

B. Parameters estimated for ebselen, in which the product concentration was measured by mass spectrometry

Figure S4: Histogram of changes in the ASE after outlier detection and refitting using the 4PL procedure

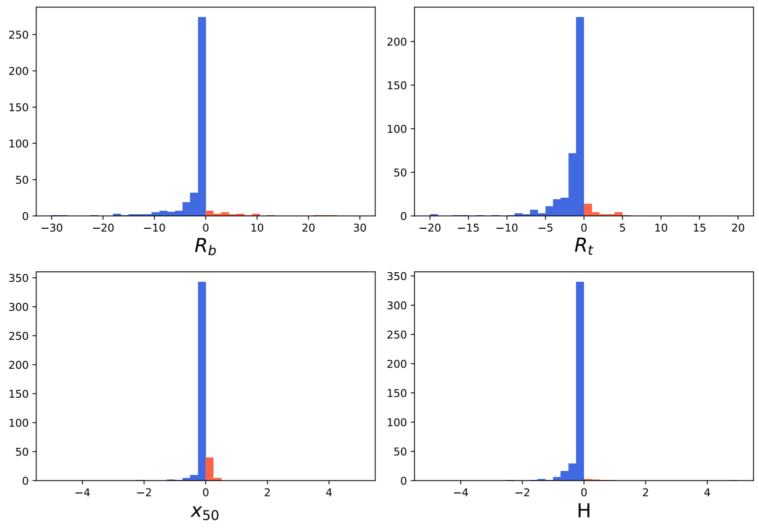

A. Histogram of the change in ASE after outlier detection and refitting using the 4PL procedure for data from the fluorescence assay. In most parameter estimates (R_b: 92.12%, R_t: 93.10%, x_{50}: 89.16%, H: 98.03%) the ASE is reduced (blue) but in some the ASE increases (orange).

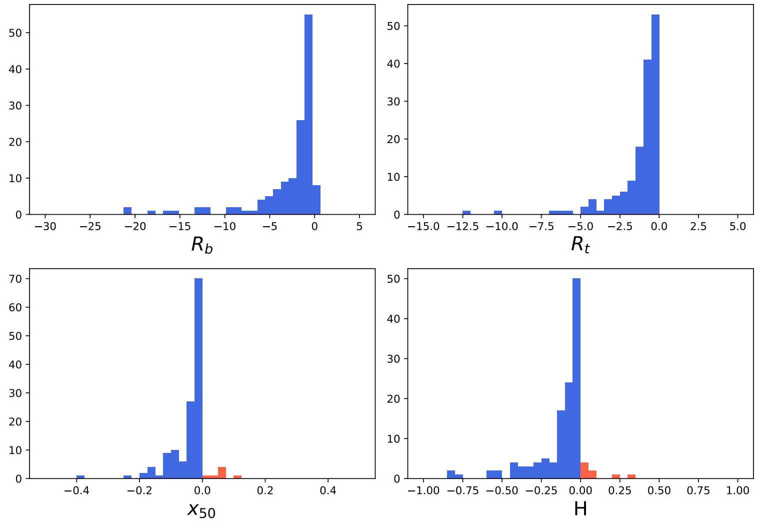

B. Histogram of the change in ASE after outlier detection and refitting using the 4PL procedure for data from the mass spectroscopy assay. In most parameter estimates (R_b: 96.67%, R_t: 100.00%, x_{50}: 92.67%, H: 91.33%), the ASE is reduced (blue) but in some the ASE increases (orange).
